# Effect of Taijiquan Exercise on Rehabilitation of Male Amphetamine-Type Addicts

**DOI:** 10.1155/2020/8886562

**Published:** 2020-11-19

**Authors:** Zhilei Zhang, Dong Zhu

**Affiliations:** ^1^College of Physical Education and Health, Heze University, Heze 274015, China; ^2^Chinese Wushu Research Center, Shanghai University of Sport, Shanghai 200438, China

## Abstract

Taijiquan is a traditional Chinese sport that is classified as a moderate exercise. Recent studies have evaluated the effectiveness of Taijiquan in substance abuse rehabilitation. *Objective*. To test the rehabilitation effect of Taijiquan exercise in patients with amphetamine (ATS) drug dependence. *Methods*. The effect of Taijiquan intervention was tested by parallel control experiment: Taijiquan group (*n* = 38) and control group (*n* = 38). The factors between the experimental groups were the group (Taijiquan group and the control group), and the factors within the group were the test time (before and after intervention). Repeated measurement analysis of variance was used to compare the two groups, and the factors that may affect the results were included in the covariance. *Results*. Taijiquan exercise promoted the balance control ability of ATS dependent patients (*p* = 0.014, *η*^2^ = 0.064), increased the overall sense of health (*p* = 0.029, *η*^2^ = 0.100), vitality (*p* = 0.030, *η*^2^ = 0.056), and mental health (*p* = 0.016, *η*^2^ = 0.061), improved trait anxiety (*p* = 0.028, *η*^2^ = 0.053), and reduced drug craving (*p* = 0.048, *η*^2^ = 0.048). *Conclusion*. Taijiquan exercise is beneficial to the physical and mental recovery of dependent patients, and the physical and mental benefits of exercise may have an effect on drug craving, which is of the most important significance for addicts to quit drugs and prevent relapse. The study is registered on the Chinese Clinical Trial Registry (No. ChiCTR1800015777).

## 1. Introduction

Amphetamine-type stimulants, referred to as ATS, are one of the most important new synthetic drugs abused by the United Nations Convention on psychotropic substances, and they are the second most used illegal drug in the world, second only to marijuana [1]. At present, methamphetamine is the most popular and most widely abused in ATS [2]. According to the report on China's drug situation in 2018 released by the Office of the China Drug Control Commission in June 2019, there are 2.404 million drug addicts nationwide, down 5.8 percent from the same period last year, but the scale is still large. Methamphetamine has replaced heroin as the “number one drug” for abuse. 1.35 million people abused methamphetamine, accounting for 56.1 percent. ATS abuse not only brings serious damage to individual physical and mental health of abusers, but also causes serious social problems. There is extensive documentation that the use of synthetic drugs is detrimental to human health. These drugs cause chronic illnesses that lead to dopaminergic neurotoxicity and cardiovascular toxicity by releasing excess stored catecholamines [3]. The release of these excess catecholamines results in serious physical and mental disorders, such as hypertension, myocardial infarction, stroke, and cardiomyopathy, as well as vascular or cerebrovascular dysfunction [4–7]. Aside from their addiction to methamphetamines, chronic abusers may also exhibit symptoms such as significant abnormal motor movement, anxiety, confusion, anhedonia, irritability, fatigue, insomnia, mood disturbances, impaired social functioning, and violent behavior [8–10]. To date, there is no effective therapy for counteracting the toxic effects of these synthetic drugs [11].

Physical exercise has long been considered important in preventing and treating several medical conditions [12]. Sports are often recommended internationally as an effective means for the treatment of physical and mental health of drug addicts [13], or as a new intervention for the treatment of drug abuse [14]. Studies have shown that exercise as an auxiliary method has a certain effect in promoting the rehabilitation of methamphetamine addicts [3]. A systematic review of 17 studies on physical exercise and drug use among adolescents showed that physical exercise reduced drug use [15]. Studies have shown that exercise is an important behavioral factor to reduce the cerebrovascular toxicity of methamphetamine [7]. Taijiquan is a traditional Chinese sport that can be classified as moderate exercise. Taijiquan has physiological and psychosocial benefits and can improve balance control, flexibility, and cardiovascular fitness of older adults with chronic conditions [16, 17]. Several studies have evaluated the effect of Taijiquan on psychological responses, such as depression, distress, well-being, life satisfaction, and perception of health [18]. Taijiquan is beneficial for both men and women over a wide age range. The American College of Sport Association recommended Taijiquan as a functional fitness exercise for improving motor skills, such as balance, coordination, gait, agility, and proprioceptive training [19]. There is evidence that Taijiquan exercise may be an effective means to help people with chronic diseases reduce stress, improve mood, or sleep [20]. The outline of the healthy China 2030 plan points out that we should support and promote traditional national and folk sports such as fitness Qigong and Taijiquan and strengthen nonmedical and health intervention and the integration of sports and medicine. The purpose of this study was to explore the rehabilitation effect of Taijiquan exercise on patients with ATS dependence, assuming that the subjects had a good improvement in physical, emotional, and drug dependence.

## 2. Methods

### 2.1. Patients

**Figure 1:**
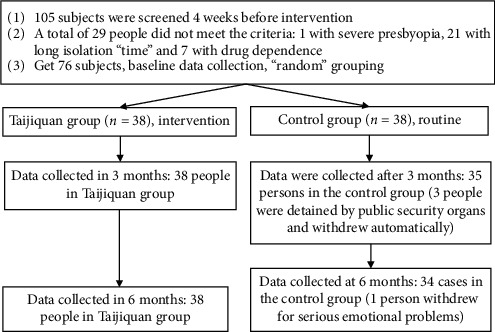
Flowchart of intervention process.

Brigade of a compulsory isolation detoxification center in Shanghai (5) cluster sampling (men): a total of 76 men were included, 38 in Shanghai and 38 outside Shanghai; the youngest was 21 years old, the oldest was 60 years old, the average was 40 years old, the minimum of BMI was 19.60, the maximum was 36.20, the average was 24.46, and the number of compulsory detoxification ranged from 1 to 7, with an average of 1.62. Inclusion criteria were (1) ATS dependence; (2) meeting the “guidelines for diagnosis and treatment of amphetamine dependence” issued by the Ministry of Health of the pPeople's Republic of China in 2009 and the evaluation criteria of addiction syndrome in “Chinese Classification and Diagnostic Criteria of Mental Disorders” (CCMD-3); (3) completing physical detoxification and entering the psychological rehabilitation stage—the isolation period is less than 12 months; (4) volunteering to participate in this researcher and having no history of Taijiquan. Rule out the following situations: having a history of mental illness, health status, or exercise taboos can not effectively complete the experiment. The data of the subjects were imported into SPSS 20.0 statistical software. They were randomly divided into Taijiquan group (experimental group, *n* = 38) and control group (routine rehabilitation group, *n* = 38). After 6 months of intervention, 38 dependent patients in the Taijiquan group and 34 dependent patients in the control group completed the established tasks. The flow chart of the Taijiquan intervention process is shown in Figure 1.

### 2.2. Taijiquan Intervention

The Taijiquan group participated in the Taijiquan exercise intervention. The Taijiquan group practiced Taijiquan once a day from Monday to Friday, including a 50-minute tutoring teaching arranged on Thursday afternoon. The tutoring teaching was completed by graduate students majoring in national traditional sports, with the technical level of six sections of Taijiquan; four 20-minute independent exercises were arranged in the evening, and the independent exercises were organized by the management police with experience in physiological rehabilitation education. The first month is teaching introduction, the last 5 months is practice, thus a total of 6 months of intervention. According to the attendance statistics of each Taijiquan exercise, the attendance rate of the Taijiquan group is more than 90%. The control group only received routine rehabilitation exercises, including the ninth set of broadcast gymnastics, sign language exercises, and queue exercises. Taijiquan group and the control group received the same routine rehabilitation exercises with the same content and time. Taijiquan group added Taijiquan exercises. According to the principles of easy to learn, easy to practice, and clear effect, the content of Taijiquan focuses on the main technical arrangement of 24-style Taijiquan.

### 2.3. Measuring Tool

Exercise intensity testing instrument: using Polar wearable team heart rate meter (model: team2; company: Boneng; origin: Finland). The monitoring results of exercise intensity showed that the average heart rate before exercise was 90.78 beats/min, 50.74% HRmax (maximum heart rate); after 5 minutes of exercise, the average heart rate increased to 99.33 beats/min, 55.52% HRmax; the peak average heart rate appeared 6 minutes after exercise, and the average peak heart rate was 113.22 beats/min, 63.28% HRmax; 5 minutes after exercise, 87.5 beats/min, 48.90%HRmax.

The physical fitness test was conducted with the National Physical Fitness Test tool, and the questionnaire was conducted with the self-made questionnaire for amphetamine addicts and international commonly used related scales, including Baker self-rating depression scale [21], state-trait anxiety scale [22], Health Status Survey (SF-36) [23], and amphetamine craving scale [24].

### 2.4. Data Collection

The blind subjects are subjects and testers, which are kept secret before the end of the intervention and until all data measurements are completed. The data of baseline (4 weeks), 3 months, and 6 months before the experiment were collected. The test site is in the physiological rehabilitation center of the compulsory isolation drug rehabilitation center. The questionnaire survey is arranged before other tests, and the questionnaire is distributed and collected by the investigator on the spot, with a recovery rate of 100%.

### 2.5. Statistical Analysis

The data were imported into SPSS 20.0 statistical software, and descriptive statistics were used for the basic situation of the subjects. Chi-square test (counting data) and independent sample *t*-test (measurement data) were used for comparison between groups. The intervention experiment was compared with the repeated measurement analysis of variance of 2 (group: Taijiquan group and control group) × 3 (test time: baseline before exercise, 3rd month, and 6th month). The frequency of admission and the number of years of dependence may have an impact on the rehabilitation effect of dependent patients, and the two factors were included in the covariance. The interactions within the group (baseline, 3 months, and 6 months), the Taijiquan group, and the control group were compared, and the physical fitness, psychological craving, depression, anxiety, and quality of life were evaluated according to the results. The spherical test was carried out before the repeated measurement analysis of variance, and the Greenhouse–Geisser method was used to correct it when it was not satisfied with the shape of the football. In the statistical analysis, if the interaction is significant, Bonferroni correction is used for posttest, simple effect analysis is carried out, and the differences between groups in different test time are compared. The index *η*^2^ (partial eta square), which reports the effect of analysis of variance, reflects the effect of the experiment.

## 3. Results

### 3.1. The Basic Situation of Taijiquan Group and Control Group

The basic characteristics of 76 amphetamine addicts included in the analysis mainly include demography, drug dependence, and so on, as shown in Table 1. The difference of admission times between the experimental group and the control group is 0.041, considering that the standard deviation of the experimental group is large, and the differences of other indicators are not significant, so the influence here can be ignored; it is considered that the baseline of the Taijiquan group and the control group is balanced.

### 3.2. Effect of Taijiquan Exercise Intervention on Physical Fitness

Physical fitness is the basic physical health condition where we should have to complete all kinds of physical activities to meet the needs of life. According to the physical fitness index measured, the national physique monitoring index used in the long-term rehabilitation evaluation of compulsory isolation detoxification center mainly includes body composition, blood pressure, and quiet pulse; the four physiques are vital capacity, grip strength, balance, and flexibility. It covers three aspects: body composition, cardiovascular system, and physique.

#### 3.2.1. Effect on Body Composition

The BMI, body fat rate, visceral fat index, and muscle percentage of ATS dependent patients were analyzed by 2 × 3 repeated measurement analysis of variance (ANOVA). The calculation results are shown in Table 2. The main effect of BMI test time was highly significant (*p* < 0.001, *η*^2^ = 0.129), but the main effect of group was not significant (*p* < 0.064, *η*^2^ = 0.041), and the interaction between time and group was not significant (*p* = 0.461, *η*^2^ = 0.008). The main effect of body fat rate test time was highly significant (*p* < 0.001, *η*^2^ = 0.349), but the main effect of group was not significant (*p* < 0.499, *η*^2^ = 0.007), and the interaction between time and group was not significant (*p* < 0.069, *η*^2^ = 0.038). The main effect of visceral fat index test time was highly significant (*p* < 0.007, *η*^2^ = 0.071), but the main effect of group was not significant (*p* = 0.690, *η*^2^ = 0.002), and the interaction between time and group was not significant (*p* = 0.060, *η*^2^ = 0.041). The main effect of muscle percentage test time was highly significant (*p* < 0.001, *η*^2^ = 0.278), but the main effect of group was not significant (*p* = 0.874, *η*^2^ < 0.001), and the interaction between time and group was not significant (*p* = 0.311, *η*^2^ = 0.017).

#### 3.2.2. Effects on Cardiovascular System

The blood pressure and heart rate of patients with amphetamine dependence were analyzed by 2 × 3 repeated measurement analysis of variance (ANOVA). The results are shown in Table 3. The main effect of systolic blood pressure test time was highly significant (*p* < 0.001, *η*^2^ = 0.149), but the main effect of group was not significant (*p* = 0.466, *η*^2^ = 0.008), and the interaction between time and group was not significant (*p* = 0.882, *η*^2^ = 0.001). The main effect of diastolic blood pressure test time was highly significant (*p* < 0.001, *η*^2^ = 0.269), but the main effect of group was not significant (*p* = 0.370, *η*^2^ = 0.012), and the interaction between time 5 and group was not significant (*p* = 0.878, *η*^2^ = 0.002). The main effect of resting heart rate test time was highly significant (*p* < 0.001, *η*^2^ = 0.154), but the main effect of group was not significant (*p* = 0.645, *η*^2^ = 0.003), and the interaction between time and group was not significant (*p* = 0.499, *η*^2^ = 0.010).

#### 3.2.3. Influence on Physique

The vital capacity, grip strength, standing on one foot (eyes closed), and body flexion of ATS dependent patients were analyzed by 2 × 3 repeated measurement analysis of variance. The results are shown in Table 4. The main effect of vital capacity test time was significant (*p* = 0.026, *η*^2^ = 0.052), but the main effect of group was not significant (*p* = 0.218, *η*^2^ = 0.022), and the interaction between time and group was not significant (*p* = 0.899, *η*^2^ = 0.002). The main effect of grip strength test time was highly significant (*p* = 0.008, *η*^2^ = 0.069), but the main effect of group was not significant (*p* = 0.300, *η*^2^ = 0.016), and the interaction between time and group was not significant (*p* = 0.647, *η*^2^ = 0.006). The main effect of standing time on one foot was highly significant (*p* < 0.001, *η*^2^ = 0.119), but the main effect of group was not significant (*p* = 0.175, *η*^2^ = 0.027), and the interaction between time and group was significant (*p* < 0.014, *η*^2^ = 0.064). Further simple effect analysis showed that there was no significant difference between the two groups at 3 months (*p* > 0.05), but there was significant difference at 6 months between the two groups (*p* < 0.05), and the standing time of Taijiquan group was longer than that of Taijiquan group. The standing time of one foot in the Taijiquan group increased all the time, while that in the control group increased at first and then decreased slightly. The main effect of sitting body flexion test time was highly significant (*p* = 0.002, *η*^2^ = 0.087), but the main effect of group was not significant (*p* = 0.145, *η*^2^ = 0.031), and the interaction between time and group was not significant (*p* = 0.427, *η*^2^ = 0.012).

### 3.3. The Influence of Taijiquan Exercise Intervention on Emotion

The results of 2 × 3 repeated test variance analysis of depression in amphetamine dependent patients showed that the main effect of depression test time was significant (*p*0.001, *η*^2^ = 0.093), but the main effect of group was not significant (*p* = 0.948, *η*^2^ < 0.001). The interaction between time and group was not significant (*p* = 0.427, *η*^2^ = 0.012). The state anxiety and trait anxiety of amphetamine addicts were analyzed by 2 × 3 repeated measurement analysis of variance (ANOVA). The main effect of time in state anxiety test was not significant (*p* = 0.470, *η*^2^ = 0.011), the main effect of group was not significant (*p* = 0.576, *η*^2^ = 0.005), and the interaction between time and group was not significant (*p* = 0.284, *η*^2^ = 0.018). The main effect of time in trait anxiety test was not significant (*p* = 0.365, *η*^2^ = 0.014), the main effect of group was not significant (*p* = 0.129, *η*^2^ = 0.034), and the interaction between time and group was significant (*p* = 0.028, *η*^2^ = 0.053). The further simple effect analysis of the interaction between trait anxiety time and group showed that there was no significant difference at 3 months (*p* = 0.098), but there was significant difference at 6 months (*p* = 0.035), and the trait anxiety decreased more in Taijiquan group. The results are shown in Table 5.

### 3.4. Effect of Taijiquan Exercise Intervention on Quality of Life

The total score and each dimension of quality of life (SF-36) of patients with amphetamine dependence were analyzed by 2 × 3 repeated measurement analysis of variance (ANOVA).The results are shown in Table 6. The main effect of SF-36 total score test time was not significant (*p* = 0.783, *η*^2^ = 0.004), the group main effect was not significant (*p* = 0.302, *η*^2^ = 0.016), and the interaction between time and group was not significant (*p* = 0.957, *η*^2^ = 0.001). The main effect of physiological function (PF) test time was not significant (*p* = 0.355, *η*^2^ = 0.015), the main effect of group was not significant (*p* = 0.731, *η*^2^ = 0.002), and the interaction between time and group was not significant (*p* = 0.394, *η*^2^ = 0.014). The main effect of physiological function (RP) test time was not significant (*p* = 0.834, *η*^2^ = 0.003), the main effect of group was not significant (*p* = 0.593, *η*^2^ = 0.004), and the interaction between time and group was not significant (*p* = 0.574, *η*^2^ = 0.008). The main effect of somatic pain (BP) test time was not significant (*p* = 0.505, *η*^2^ = 0.010), the main effect of group was not significant (*p* = 0.943, *η*^2^ < 0.001), and the interaction between time and group was not significant (*p* = 0.271, *η*^2^ = 0.019). The main effect of (GH) test time of general sense of health was not significant (*p* = 0.276, *η*^2^ = 0.038), the main effect of group was not significant (*p* = 0.267, *η*^2^ = 0.018), and the interaction between time and group was significant (*p* = 0.029, *η*^2^ = 0.100). Further simple effect analysis showed that there was no significant difference at 3 months (*p* = 0.260), but there was significant difference at 6 months (*p* < 0.01). The overall sense of health in Taijiquan group was significantly higher than that in the control group. The main effect of life vitality (VT) test time was not significant (*p* = 0.789, *η*^2^ = 0.003), the main effect of group was not significant (*p* = 0.115, *η*^2^ = 0.036), and the interaction between time and group was significant (*p* = 0.030, *η*^2^ = 0.056). Further simple effect analysis showed that there was significant difference between 3 months and 6 months (*p* < 0.05). The vitality (VT) of Taijiquan group was significantly higher than that of the control group. The main effect of social function (SF) test time was highly significant (*p* = 0.002, *η*^2^ = 0.085), but the main effect of group was not significant (*p* = 0.735, *η*^2^ = 0.002), and the interaction between time and group was not significant (*p* = 0.192, *η*^2^ = 0.024). The main effect of affective function (RE) test time was not significant (*p* = 0.659, *η*^2^ = 0.006), the main effect of group was not significant (*p* = 0.251, *η*^2^ = 0.019), and the interaction between time and group was not significant (*p* = 0.178, *η*^2^ = 0.025). The main effect of mental health (MH) test time was not significant (*p* = 0.205, *η*^2^ = 0.023), the main effect of group was not significant (*p* = 0.151, *η*^2^ = 0.030), and the interaction between time and group was significant (*p* = 0.016, *η*^2^ = 0.061). Further simple effect analysis showed that there was no significant difference at 3 months (*p* < 0.05), but there was significant difference at 6 months (*p* < 0.05). The mental health (MH) of Taijiquan group was significantly higher than that of the control group.

### 3.5. Effect of Taijiquan Exercise Intervention on Drug Craving

A 2 × 3 repeated measurement analysis of variance for ATS dependent patients' psychological craving for (DSQ) was carried out, and the results are shown in Table 7. The main effect of psychological craving (DSQ) test time was not significant (*p* = 0.544, *η*^2^ = 0.008), the main effect of group was not significant (*p* = 0.052, *η*^2^ = 0.055), and the interaction between time and group was significant (*p* = 0.048, *η*^2^ = 0.048). Simple effect analysis showed that the difference was significant at 3 months (*p* = 0.016) and highly significant at 6 months (*p* = 0.008). The psychological craving of Taijiquan group was much lower than that of the control group.

## 4. Discussion

The standing time of one foot in the Taijiquan group has been increasing, while that in the control group increased at first and then decreased slightly. The balance ability of the Taijiquan group was improved, which is of great significance to the work and life of the dependent subjects. A foreign meta-analysis summarized 7 randomized controlled trials of Taijiquan, including 1088 participants (544 subjects and 544 control subjects). The results show that Taijiquan exercise can improve flexibility and standing time on one leg, which helps to improve balance control, which may prevent falls [25]. Compared with other intervention or nontreatment, Taijiquan is more effective in preventing falls in frail and high-risk adults [26]. It is worth noting that the values of BMI in both groups were obese at each time point (BMI > 24), which showed an increasing trend as a whole, while the visceral fat index increased more significantly. Taijiquan exercise has a positive effect on BMI, body fat rate, visceral fat index, and muscle percentage, but the difference is not significant. The test time was 3 months in summer July, and the average daytime temperature in Shanghai was 30°C or above, up to 40°C. Dependent people generally reflect poor appetite, which may be an important factor in the decrease of BMI and other indicators, but also in line with the normal law of people's life. There is no difference in vital capacity between the Taijiquan group and the control group, which may be related to the small amount of exercise and exercise intensity.

Taijiquan exercise intervention reduced the subjects' anxiety; many experiments reported similar views. It has been reported that moderate intensity aerobic exercise can significantly reduce the anxiety and depression of methamphetamine addicts [27]. Taijiquan exercise is beneficial to the rehabilitation of cardiovascular diseases; at the same time, it can reduce anxiety and improve mood. Taijiquan therapy is considered to be one of the important choices of rehabilitation treatment [28]. The benefit of Taijiquan on mood improvement has gradually attracted people's attention. More and more scientific evidence shows that Taijiquan exercise is beneficial to improve mental and emotional health and reduce stress [18]. Taijiquan provides a possible way to reduce anxiety [29]. Long-term exercise in the form of collective organization can better relieve anxiety, especially trait anxiety [30]. Taijiquan may provide an opportunity to reconnect your body and mind. Taijiquan pays attention to the inner mental activities and pays attention to “intention without exertion.” The mental interpretation of the thirteen potentials says, “the mind is the order, and the qi is the flag; first in the mind, then in the body.” Chen Xin said, “the heart of boxing is the master; it is used in the heart; this is the true formula; the operation is all in one mind.” Dependent people can achieve physical and mental balance through Taijiquan exercise and achieve a better level of social function. Karen Horney, a German-American psychologist and psychiatrist, believes that drug abuse can be understood as an individual's escape strategy to certain social situations, and the psychological defense process of personal anxiety is a kind of “transfer.” Indulging in drug use can get rid of or avoid anxiety [23]. Drug use can improve negative emotions such as anxiety, depression, and despair, so some people tend to abuse drugs. Most drug abusers have obvious mental disorders and psychological problems, lack trust in others and society, have empty life, and escape through drug abuse. Although the reasons for this phenomenon are very complicated, drug abuse is obviously a comprehensive physiological and behavioral response of addicts to seek advantages and avoid disadvantages [18]. The communication between human mind and body is very complex, and when such abuse is carried out for a long time, it becomes a dependent behavior of euphoria when it replaces the original brain natural reward or shows as a new reward. Taijiquan intervention makes the trait anxiety of dependent patients in Taijiquan group decrease more, which has important rehabilitation significance, but although the depression level of dependent patients decreases greatly, the effect of Taijiquan group and control group is not obvious. Exercise tried in the process of clinical rehabilitation shows that high-intensity training seems to be particularly beneficial to depressive symptoms [31], which seems to be an experimental direction.

Taijiquan exercise has an obvious effect on improving the quality of life, and the overall sense of health, vitality, and mental health in the Taijiquan group are significantly higher than those in the control group. Some studies have conducted a randomized controlled trial of Taijiquan, which shows that Taijiquan is effective in managing mental health related to cancer, especially in reducing physical and mental fatigue and discomfort and increasing vitality [32]. Taijiquan practice requires the elimination of distractions, concentration, and calmness of mind. This process is actually a process of psychological adjustment and exercise. In addition, the psychological promotion benefit of Taijiquan should have a synergistic effect with traditional music. Taijiquan exercise is usually accompanied by quaint and leisurely music. In addition, Taijiquan exercises are mostly group activities and group communication activities, which can promote people's socialization and interpersonal relationship development and improve interpersonal communication, emotional communication, and self-closure in sports, so as to regulate and dredge negative emotions and enhance the sense of health. Quality of life is the subjective feeling and satisfaction of individuals in physical, psychological, and social aspects. Compared with epidemiological indicators such as morbidity, quality of life can sometimes more accurately reveal the interaction between health and disease, life events, and environment. Studies have shown that the quality of life is related to the cure of many diseases, and improving the quality of life of patients has become a problem that can not be ignored in the process of treatment [33]. Drug addicts are more addicted to the illusory world and the feelings brought by drugs, self-closure, closed circles, and reduced communication and communication with the outside world; normal living habits and social activities disappear, not to mention the social barriers. Taijiquan exercise provides a way to socialize communication.

The psychological craving in the Taijiquan group is much lower than that in the control group, which may be due to the fact that Taijiquan promotes the decline of craving by acting on other intermediary factors. Animal experiments show that physical activity reduces the self-administration of cocaine in animals, and physical activity may be an effective intervention in drug abuse prevention planning [34]. Physical activity, to some extent, is related to well-being, which is similar to drug-induced addictive behavior. Animal experiments have shown that running and cocaine have a common induction mechanism in the brain's reward pathway [35, 36]. Exercise can be used as an alternative nondrug reward to compete with drugs and reduce the possibility of their use [37]. At present, there are many theories about how exercise produces beneficial effects; although it is not entirely clear, but it can be determined that physical activity can be used as a preventive intervention for drug abuse by improving stress response and emotional regulation [38]. When abstaining from drug abuse, physical exercise can be a good medicine to enhance the function of the brain [39]. By participating in physical exercise, dependent people can improve cognitive control functions such as brain memory lost by drug abuse. According to the degree of muscle participation in exercise, Taijiquan is a sports skill with complex spatial navigation. During this exercise, practitioners need to integrate all kinds of self-ontological spatial information and participate in spatial navigation and spatial information at the same time. Taijiquan exercise seems to be more effective in improving the cognitive ability of people who depend on ATS. Therefore, Taijiquan exercise can improve the cognitive control system of ATS dependent patients, inhibit their decision to use illegal drugs, and play a good role in protecting long-term abstinence. The reduction of the psychological craving of the dependent is of great significance to the return to society. There is a certain relationship between psychological craving and dependence years, emotion, and physical and mental health [40], so it is of practical significance to abstain from treatment as soon as possible, scientifically increase physical exercise, and improve mental health. The mechanism of Taijiquan reducing psychological craving of ATS dependent patients needs to be further confirmed.

The exercise program studied pays too much attention to the control of exercise risk, limits the increase of exercise intensity and amount of exercise, and may affect the performance of exercise efficiency. If the dependent patients are followed up after leaving the institute, it is a good topic to observe the long-term effects of exercise habits. There are a large number of dependent people for community detoxification; they are closer to the natural state of society. Taijiquan intervention in the community may be more promising.

## 5. Conclusions

The balance ability, trait anxiety, overall health, vitality, and mental health of the ATS dependent Taijiquan group were significantly better than those of the control group. These factors may indirectly affect their psychological craving for drugs, and the psychological craving of the Taijiquan group is much lower than that of the control group. The decrease of psychological craving for drugs is of great significance for them to return to society.

## Figures and Tables

**Table 1:** Comparison of the basic conditions between the Taijiquan group and the control group (*n* = 76, *M* ± SD).

Participants	Taijiquan group (*n* = 38)	Control group (*n* = 38)	*t*/*χ*^2^	*p*
Age	41.08 ± 9.94	39.11 ± 8.90	0.912	0.365
Height (m)	1.72 ± 0.47	1.73 ± 0.54	−0.906	0.368
Weight (kg)	72.10 ± 7.80	73.60 ± 9.11	−0.770	0.444
Monthly frequency	12.71 ± 16.35	10.68 ± 10.24	0.647	0.519
Dependence (age)	7.76 ± 5.90	7.13 ± 5.39	0.487	0.627
Hypertension			0	1
Yes	9(23.7%)	9(23.7%)		
No	29(76.3%)	29(76.3%)		
Use of drugs			0.060	0.807
One person	13 (34.2 %)	12 (32.9 %)		
2 or more	25(65.8%)	26(67.1%)		
The number of times of compulsory detoxification	1.89 ± 1.39	1.34 ± 0.85	2.092	0.041^*∗*^
Monthly cost of medication	2505.26 ± 2671.67	3044.74 ± 3489.73	−0.757	0.452

*Note*. ^*∗*^*p* < 0.05.

**Table 2:** Comparison of the changes of body composition between the two groups of dependent subjects before and after the experiment (*M* ± SD).

	Taijiquan group (*n* = 38)			Control group (*n* = 34)			Time F value	Group F value	Time × group F value
	Baseline	3 months	6 months	Baseline	3 months	6 months			
BMI (kg/m^2^)	24.34 ± 2.22	24.20 ± 2.49	24.85 ± 2.36	24.66 ± 2.81	24.41 ± 2.67	25.43 ± 2.63	10.095^*∗∗*^	0.549	2.924
Body fat percentage (%)	21.43 ± 3.30	20.89 ± 3.52	23.95 ± 3.00	21.70 ± 3.17	20.77 ± 3.37	24.61 ± 3.30	36.481^*∗∗*^	0.463	2.720
Visceral fat index	9.52 ± 2.80	9.49 ± 3.10	10.26 ± 2.97	9.62 ± 3.40	9.31 ± 3.22	10.74 ± 3.26	5.202^*∗∗*^	0.161	2.875
Muscle percentage (%)	32.66 ± 1.61	33.08 ± 1.80	31.60 ± 1.53	32.75 ± 1.63	33.15 ± 1.67	31.48 ± 1.74	26.158^*∗∗*^	0.025	1.149

*Note*. ^*∗∗*^*p* < 0.01.

**Table 3:** Comparison of the changes of blood pressure and resting heart rate between the two groups of dependent subjects before and after the experiment (*M* ± SD).

	Taijiquan group (*n* = 38)			Control group (*n* = 34)			Time F value	Group F value	Time × group F value
	Baseline	3 months	6 months	Baseline	3 months	6 months			
Systolic blood pressure (mmHg)	124.67 ± 14.10	119.58 ± 15.93	131.28 ± 18.49	128.19 ± 13.26	122.10 ± 14.66	134.35 ± 17.38	11.943^*∗∗*^	0.538	0.089
Diastolic blood pressure (mmHg)	75.53 ± 9.85	74.50 ± 10.76	84.41 ± 8.01	78.41 ± 9.51	76.37 ± 10.93	86.91 ± 11.28	25.070^*∗∗*^	0.815	0.130
Heart rate (/min)	71.71 ± 10.04	64.66 ± 10.33	70.53 ± 8.60	71.31 ± 8.49	65.72 ± 6.52	73.51 ± 11.12	12.422^*∗∗*^	0.214	0.699

*Note*. ^*∗∗*^*p* < 0.01.

**Table 4:** Comparison of the changes of physique indexes between the two groups of dependent patients before and after the experiment (*M* ± SD).

	Taijiquan group (*n* = 38)			Control group (*n* = 34)			Time F value	Group F value	Time × group F value
	Baseline	3 months	6 months	Baseline	3 months	6 months			
Vital capacity (ml)	2565.89 ± 704.31	2616.76 ± 663.68	2792.74 ± 698.13	2704.41 ± 675.92	2819.53 ± 644.58	2994.35 ± 464.05	3.735^*∗*^	1.545	0.118
Grip strength (kg)	51.03 ± 7.39	51.91 ± 7.03	47.98 ± 7.03	52.30 ± 8.77	52.64 ± 5.66	49.86 ± 6.90	5.015^*∗∗*^	1.090	0.437
Standing on one foot (s)	10.82 ± 7.45	18.29 ± 12.67	24.61 ± 20.13	12.03 ± 8.60	17.02 ± 12.55	16.54 ± 9.29	9.183^*∗∗*^	1.875	4.682^*∗∗*^
Body flexion (cm)	2.02 ± 7.78	5.29 ± 7.57	7.69 ± 6.64	5.31 ± 7.85	8.36 ± 8.49	9.05 ± 9.03	6.448^*∗∗*^	2.178	0.855

*Note*. ^*∗*^*p* < 0.05, ^*∗∗*^*p* < 0.01.

**Table 5:** Comparison of the changes of emotion between the two groups of dependent patients before and after the experiment (*M* ± SD).

	Taijiquan group (*n* = 38)			Control group (*n* = 34)			Time F value	Group F value	Time × group F value
	Baseline	3 months	6 months	Baseline	3 months	6 months			
BDI	9.11 ± 5.24	8.13 ± 5.86	5.71 ± 4.75	9.00 ± 5.15	8.06 ± 5.06	6.85 ± 6.45	6.992^*∗∗*^	0.004	0.857
S-AI	44.61 ± 10.04	41.47 ± 10.77	39.26 ± 10.91	44.71 ± 9.31	43.97 ± 10.15	43.00 ± 10.53	0.760	0.315	1.272
T-AI	45.47 ± 8.25	41.66 ± 9.63	38.71 ± 11.56	46.09 ± 8.93	46.18 ± 9.31	45.76 ± 9.61	0.999	2.358	3.821^*∗*^

*Note*. ^*∗*^*p* < 0.05, ^*∗∗*^*p* < 0.01, BDI: Baker self-rating depression scale, S-AI: state anxiety, and T-AI: trait anxiety.

**Table 6:** Comparison of the changes of quality of life between the two groups of dependent patients before and after the experiment (*M* ± SD).

	Taijiquan group (*n* = 38)			Control group (*n* = 34)			Time F value	Group F value	Time × group F value
	Baseline	3 months	6 months	Baseline	3 months	6 months			
SF-36	580.58 ± 107.01	586.61 ± 128.54	609.79 ± 119.71	604.65 ± 127.00	600.79 ± 110.76	620.97 ± 96.73	0.245	1.081	0.044
PF	90.66 ± 7.64	83.16 ± 16.42	87.50 ± 13.99	88.38 ± 14.81	85.00 ± 16.05	89.41 ± 14.71	1.045	0.119	0.938
RP	87.50 ± 28.32	83.68 ± 27.23	88.82 ± 27.07	84.56 ± 31.39	85.29 ± 26.91	92.65 ± 20.90	0.182	0.288	0.557
BP	85.37 ± 18.62	83.00 ± 23.77	83.71 ± 21.81	84.53 ± 21.34	81.38 ± 19.82	87.94 ± 15.42	0.686	0.005	1.318
GH	67.32 ± 19.78	71.34 ± 24.71	77.58 ± 23.02	68.44 ± 23.00	64.18 ± 20.15	65.59 ± 19.01	0.886	1.253	4.015^*∗*^
VT	64.16 ± 18.08	68.66 ± 19.35	70.84 ± 18.00	65.06 ± 20.97	59.32 ± 17.03	59.62 ± 15.31	0.173	2.544	4.008^*∗*^
SF	54.89 ± 23.96	59.21 ± 16.61	58.18 ± 20.00	54.09 ± 15.04	61.76 ± 17.93	50.78 ± 14.76	6.326^*∗∗*^	0.116	1.672
RE	84.21 ± 31.71	79.82 ± 32.46	84.21 ± 34.43	82.35 ± 36.00	88.24 ± 28.29	92.16 ± 24.70	0.418	1.338	1.747
MH	60.58 ± 19.40	66.58 ± 20.78	72.37 ± 18.59	61.24 ± 16.80	57.68 ± 14.62	60.82 ± 14.79	1.614	2.113	4.433^*∗*^

*Note*. ^*∗*^*p* < 0.05, ^*∗∗*^*p* < 0.01; SF-36: total score of quality of life; PF: physiological function; RP: physiological function; BP: body pain; GH: general sense of health; VT: vitality; SF: social function; RE: emotional function; MH: mental health.

**Table 7:** Comparison of the changes of psychological craving (DSQ) between the two groups of dependent patients before and after the experiment (*M* ± SD).

	Taijiquan group (*n* = 38)			Control group (*n* = 34)			Time F value	Group F value	Time × group F value
	Baseline	3 months	6 months	Baseline	3 months	6 months			
DSQ	93.79 ± 41.98	73.42 ± 28.95	69.03 ± 24.00	91.74 ± 19.70	88.50 ± 29.66	83.06 ± 18.93	0.531	3.926	3.419^*∗*^

注: ^*∗*^*p* < 0.05; DSQ: psychological craving.

## Data Availability

The data used to support the findings of this study are available from the corresponding author upon request.
